# Viral Infection Is Not Uncommon in Adult Patients with Severe Hospital-Acquired Pneumonia

**DOI:** 10.1371/journal.pone.0095865

**Published:** 2014-04-21

**Authors:** Hyo-Lim Hong, Sang-Bum Hong, Gwang-Beom Ko, Jin Won Huh, Heungsup Sung, Kyung-Hyun Do, Sung-Han Kim, Sang-Oh Lee, Mi-Na Kim, Jin-Yong Jeong, Chae-Man Lim, Yang Soo Kim, Jun Hee Woo, Younsuck Koh, Sang-Ho Choi

**Affiliations:** 1 Department of Infectious diseases, Asan Medical Center, University of Ulsan College of Medicine, Seoul, Republic of Korea; 2 Department of Pulmonary and Critical Care Medicine, Asan Medical Center, University of Ulsan College of Medicine, Seoul, Republic of Korea; 3 Department of Laboratory Medicine, Asan Medical Center, University of Ulsan College of Medicine, Seoul, Republic of Korea; 4 Department of Radiology, Asan Medical Center, University of Ulsan College of Medicine, Seoul, Republic of Korea; 5 Asan Institute for Life Sciences, Asan Medical Center, University of Ulsan College of Medicine, Seoul, Republic of Korea; University Hospital San Giovanni Battista di Torino, Italy

## Abstract

**Background:**

Viral pathogens have not generally been regarded as important causes of severe hospital-acquired pneumonia (HAP), except in patients with hematologic malignancy or transplant recipients. We investigated the role and distribution of viruses in adult with severe HAP who required intensive care.

**Methods:**

From March 2010 to February 2012, adult patients with severe HAP required admission to the intensive care unit (ICU), 28-bed medical ICU in a tertiary care hospital, were prospectively enrolled. Respiratory viruses were detected using multiplex reverse-transcription polymerase chain reaction and/or shell vial culture.

**Results:**

A total of 262 patients were enrolled and 107 patients (40.8%) underwent bronchoscopic BAL for etiologic diagnosis. One hundred and fifty-six patients (59.5%) had bacterial infections and 59 patients (22.5%) had viral infections. Viruses were detected in BAL fluid specimens of 37 patients (62.7%, 37/59). The most commonly identified viruses were respiratory syncytial virus and parainfluenza virus (both 27.1%, 16/59), followed by rhinovirus (25.4%, 15/59), and influenza virus (16.9%, 10/59). Twenty-one patients (8.0%, 21/262) had bacterial-viral coinfections and *Staphylococcus aureus* was the most commonly coexisting bacteria (n = 10). Viral infection in non-immunocompromised patients was not uncommon (11.1%, 16/143), although it was not as frequent as that in immunocompromised patients (36.4%, 43/119). Non-immunocompromised patients were significantly older than immunocompromised patients and had significantly higher rates of underlying chronic obstructive pulmonary disease, tuberculous destroyed lung and chronic kidney disease. The 28 day mortalities of patients with bacterial infections, viral infections and bacterial-viral coinfections were not significantly different (29.5%, 35.6% and 19.0%, respectively; *p = *0.321).

**Conclusions:**

Viral pathogens are not uncommon in adult patients with severe HAP who required ICU admission. Since viral pathogens may cause severe HAP and could be a potential source of viral transmission, further investigation is required to delineate the role of viral pathogens in severe HAP.

## Introduction

Hospital-acquired pneumonia (HAP) is the second most common nosocomial infection, and severe HAP requiring treatment in the intensive care unit (ICU) is associated with high morbidity and mortality [1], [2]. Bacterial pathogens are well known as principal causes of HAP. Such species include *Staphylococcus aureus,* including methicillin-resistant *S. aureus* (MRSA), *Pseudomonas aeruginosa, Acinetobacter* species, *Klebsiella pneumonia,* and *Escherichia coli* [3], [4]. Traditionally, respiratory viruses have been given minimal attention as a cause of hospital-acquired infection [5]. Although several previous investigators reported on the role of respiratory viruses, those studies were mainly confined to patients with hematologic malignancy [6], or hematopoietic stem cell or solid organ transplant recipients [7]–[13]. Also, these studies included both upper respiratory tract infections and lower respiratory tract infections, and did not distinguish between hospital-acquired and community-acquired infections. Hospital-acquired respiratory viral infections, including influenza virus [14], [15], human respiratory syncytial virus [16], [17], human metapneumovirus [18], and SARS-coronavirus [19] outbreaks, have been demonstrated previously in non-immunocompromised patients as well as immunocompromised patients.

In recent years, the use of molecular diagnostic methods, especially the polymerase chain reaction (PCR) assays, has improved the ability to detect respiratory viruses in clinical specimens, including newly discovered respiratory viruses such as human metapneumovirus, human coronaviruses NL63 and HKU1, and bocavirus [20], [21]. Several previous investigations that compared PCR with conventional methods (viral culture, antigen detection, and serological assays) for virus identification showed that the incidence of viral infection has been considerably underestimated in the absence of PCR [9], [10], [22]. Using multiplex reverse-transcription PCR methods, we previously showed that viral infection is as common as bacterial infection in adult patients from the community with severe pneumonia requiring ICU admission [23].

Excluding influenza infection, to the best of our knowledge, no investigation has been focused on the role of respiratory viruses as a cause of HAP. The greater understanding of the distribution and role of viral infection might provide new insight into HAP. The objective of the current study was to investigate the incidence and distribution of viruses in adult patients with severe HAP requiring ICU admission.

## Methods

### Ethics Statement

The need for informed consent was waived in view of the observational nature of the study with no interventions performed. The protocol and standardized clinical form, including the waiver of informed consent, were approved by the Asan Medical Center Institutional Review Board (IRB number: 2010-0079).

### Study Setting

The study was performed at a medical ICU of the Asan Medical Center, a tertiary referral hospital in Seoul, Republic of Korea. This university-affiliated teaching hospital has 2700 beds and eight ICUs. During the study period, most of the adult patients with severe HAP requiring ICU care were referred to the medical ICU. The medical ICU is a closed 28-bed unit managed by five board-certified intensivists. All intensivists attend structured twice daily bedside rounds. Fiberoptic bronchoscopy with bronchoalveolar lavage (BAL) was preferably performed on patients with bilateral interstitial pattern infiltration or non-resolving pneumonia, at the discretion of the physician’s judgment. The BAL protocol has been described in detail elsewhere [23].

### Study Design, Patient Population, and Data Collection

This was prospective cohort study conducted during the 2 year period between March, 2010 and February, 2012. All consecutive adult patients with severe HAP were included in the study. The investigators prospectively collected data on patient demography, comorbidities, smoking habit, laboratory data including microbiologic tests, radiologic findings, clinical features, length of ICU and hospital stay, and outcome. Disease severity was evaluated by the Acute Physiological and Chronic Health Evaluation (APACHE) II and the Sequential Organ Failure Assessment (SOFA) score, and the underlying disease was classified according to McCabe and Jackson classification [24]. Since we included patients with severe HAP only in our present study cohort, none of these current patients overlapped with those in our prior study, which analyzed patients with severe CAP or HCAP [23].

### Definitions

HAP was defined according to American Thoracic Society/Infectious Diseases Society of America guidelines [4], [25], which included the presence of a new and persistent radiographic infiltrate occurring 48 hours or more after admission, plus two or more of the following: *(i)* fever (38.5°C or higher) or hypothermia (<36.5°C); *(ii)* leukocytosis (white blood cells >10,000/mm^3^ or <4,000/mm^3^); or *(iii)* purulent tracheal aspirate or sputum. Pneumonia was classified as severe in patients who required mechanical ventilation or were in a state of septic shock with the need for vasopressors at ICU admission [26]. Patients were classified as immunocompromised if they: *(i)* were administered corticosteroids daily (at least 5 mg per day of prednisolone or an equivalent drug), *(ii)* were seropositive for human immunodeficiency virus, *(iii)* had received a solid organ or hematopoietic stem cell transplant, *(iv)* had received treatment with radiation therapy or chemotherapy for an underlying malignancy during the 6 months prior to hospital admission, or *(v)* had an underlying acquired immune deficiency disorder [27].

### Microbiological Evaluation

Microbiological evaluations were conducted according to standard procedure as previously described [23]. These included three sets of blood culture, gram stain and culture of sputum or endotracheal aspirates, or BAL fluid, and urinary antigen testing for *Streptococcus pneumoniae* and *Legionella pneumophila* (Binax Inc., Portland, Maine, USA). PCR was used to detect atypical bacterial pathogens such as *L. pneumophila, Mycoplasma pneumonia* and *Chlamydophila pneumoniae*, using the BD ProbeTec ET Atypical Pneumonia Assay (Becton Dickinson, Sparks, USA). Microorganisms identified from specimens which had been collected within 72 hours after the diagnosis of pneumonia were included as pathogens.

Respiratory viruses were tested by multiplex reverse-transcription PCR (RT-PCR) assay, using a Seeplex 15RV ACE Detection kit (Seegene Inc., Seoul, Korea), for influenza A and B viruses, adenovirus, parainfluenza virus types 1, 2, 3, and 4, respiratory syncytial virus types A and B, rhinovirus, human metapneumovirus, enterovirus, human coronaviruses OC43, 229E/NL63, and HKU1, and bocavirus [21], [23], [28]. Shell vial culture was performed for influenza virus, parainfluenza virus, respiratory syncytial virus, cytomegalovirus, and adenovirus (Diagnostic Hybrids, Inc., Athens, USA) using BAL samples only.

### Statistical Analysis

Data were analysed using IBM SPSS for Windows (version 19.0; IBM Corp., Armonk, NY, USA). Results are expressed as the mean ± _SD_, or median (interquartile range (IQR)). Either the chi-square test or Fisher’s exact test was used to compare categorical variables, and the Student’s *t* test or the Mann-Whitney *U* test was used to compare continuous variables. Variables that had presented a *p* value of less than 0.2 in the univariate analysis were included in the multiple logistic regression model. The results were expressed as adjusted odds ratios (aORs) with 95% confidence intervals (CIs). A *p* value of less than 0.05 was considered statistically significant.

## Results

### Patient Characteristics

During the study period, a total of 1854 patients were admitted to our medical ICU and 722 patients were diagnosed with pneumonia. Of these, 279 had HAP, 156 had community-acquired pneumonia (CAP) and 287 had healthcare-associated pneumonia (HCAP). After excluding 17 HAP patients who did not meet the criteria for severe pneumonia, a total of 262 patients with severe HAP were finally included for analysis.

The baseline clinical characteristics and underlying diseases are listed in Table 1. The mean age was 64.5±13.5 years and the median length of hospital stay prior to pneumonia diagnosis was 20 days (IQR, 10−42). The main underlying diseases were hematologic malignancy (29.0%, n = 76), diabetes mellitus (29.0%, n = 76), structural lung disease (22.9%, n = 60) and solid cancer (22.5%, n = 59). Of these, 119 patients (45.4%) were categorized as in an immunocompromised state: 57 (47.9%) had recently received chemotherapy and/or radiation therapy, 56 (47.1%) were receiving corticosteroid therapy, 33 (27.7%) were administered non-steroidal immunosuppressants, 20 (16.8%) received bone marrow transplantation, and 10 (8.4%) received solid organ transplantation (Table S1). The mean APACHE II was 25.4±6.8 and the mean SOFA score was 10.0±3.5. A total of 251 patients (95.8%) underwent mechanical ventilation and in 123 (46.9%) patients, clinical presentation was septic shock at the time of ICU admission.

**Table 1:** Demographics, underlying disease/condition, and clinical characteristics of patients with severe hospital-acquired pneumonia.

	HAP patients (n = 262)
Male, n (%)	191 (72.9)
Mean age (SD), y	64.5 (13.5)
Hospital stay prior to HAP, median (IQR), d	20 (10–42)
Underlying diseases or conditions, n (%)	
Hematologic malignancy	76 (29.0)
Diabetes mellitus	76 (29.0)
Structural lung disease	60 (22.9)
Chronic obstructive pulmonary disease	28 (10.7)
Interstitial lung disease	19 (7.3)
Tuberculous destroyed lung	17 (6. 5)
Bronchiectasis	4 (1.5)
Solid cancer	59 (22.5)
Cerebrovascular attack	21 (8.0)
Heart failure	21 (8.0)
Bone marrow transplantation	20 (7.6)
Liver cirrhosis	12 (4.6)
Alcoholism	10 (3.8)
End-stage renal disease	10 (3.8)
Solid organ transplantation	10 (3.8)
Chronic renal failure	8 (3.1)
Immunocompromised state^a^	119 (45.4)
Receipt of immunosuppressive therapy	64 (24.4)
Receipt of recent chemotherapy (within 1 month)	57 (21.8)
Neutropoenia (absolute neutrophil count <500/mm^3^)	47 (17.9)
Recent surgery (within 1 month)	40 (15.3)
Active smoker	13 (5.0)
APACHE II score (SD)	25.4 (6.8)
SOFA score (SD)	10.0 (3.5)
Mechanical ventilation, n (%)	251 (95.8)
Septic shock at admission, n (%)	123 (46.9)

HAP = hospital-acquired pneumonia; APACHE = Acute Physiology and Chronic Health Evaluation; SOFA = Sequential Organ Failure Assessment.

a The immunocompromised state was verified if the patients: *(i)* had daily administration of corticosteroids (at least 5 mg per day of prednisolone or an equivalent drug), *(ii)* were solid organ or hematopoietic stem cell transplant recipients, *(iii)* had received treatment with chemotherapy for an underlying malignancy during the 6 months prior to ICU admission, and *(iv*) had an underlying acquired immune deficiency disorder.

### Distribution of Identified Respiratory Pathogens

**Figure 1:**
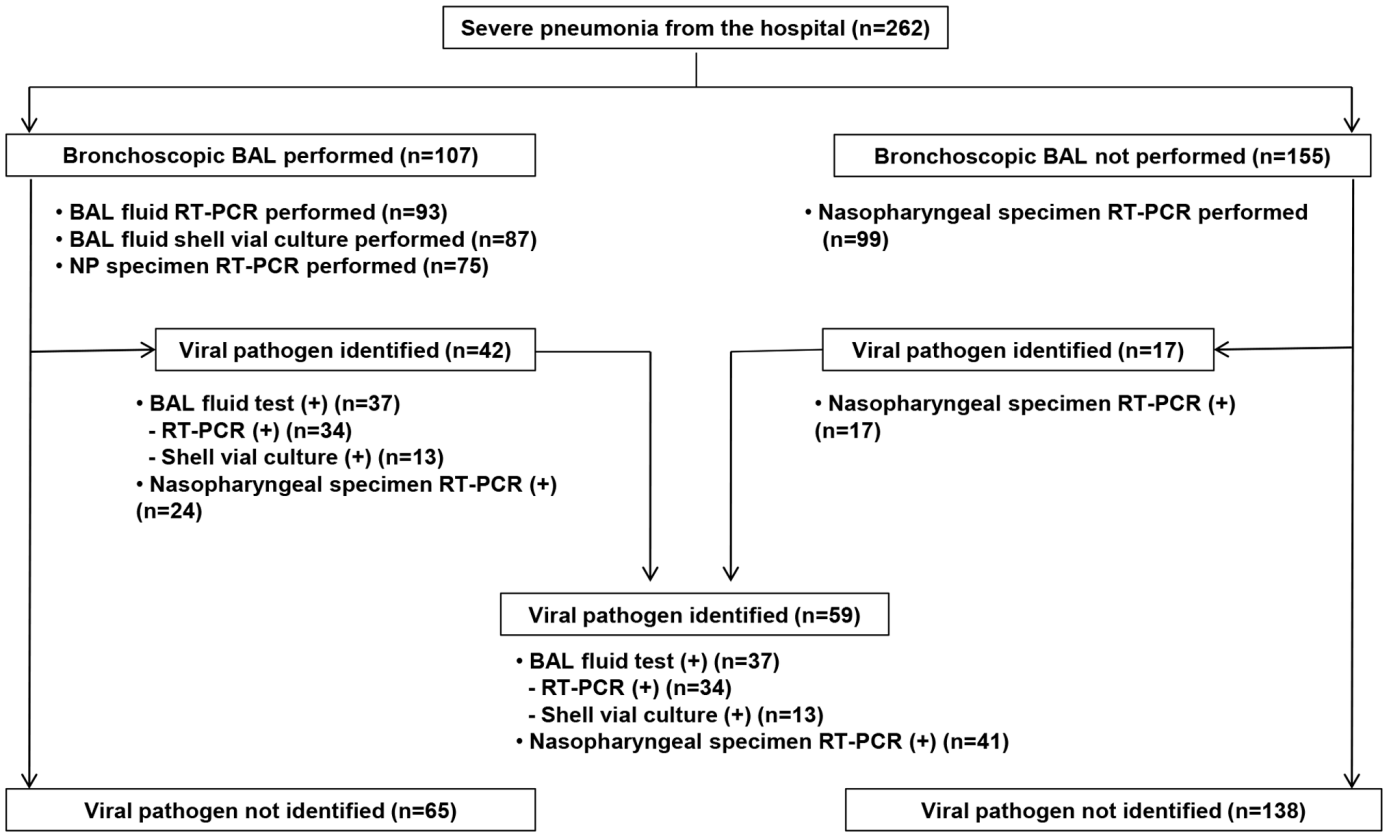
Flow diagram of the virus identification process in 262 patients with severe hospital-acquired pneumonia.

One or more respiratory pathogens were determined in 201 (76.7%) of 262 patients, and 107 patients (40.8%) underwent bronchoscopic BAL for etiologic diagnosis (Figure 1). The distribution of respiratory pathogens is shown in Table 2. Bacterial infections were observed in 156 patients (59.5%). Of these, 130 (49.6%, 130/262) were diagnosed solely with bacterial infection. Viral infections were found in 59 patients (22.5%). Of these, 31 (11.8%, 31/262) were diagnosed solely with viral infections. The coinfected patients included 21 (8.0%, 21/262) with bacterial-viral coinfections and seven (2.7%, 7/262) with viral-fungal coinfections. Viral infections were found in 36.1% (43/119) of immunocompromised patients and 11.2% (16/143) of non-immunocompromised patients (*p*<0.001). The identification of viral pathogens in accordance with the immunocompromised conditions is summarized in Table S1.

**Table 2:** Identity of pathogens in patients with severe hospital-acquired pneumonia^a^.

	HAP patients (n = 262) (%)
**No detected pathogen**	61 (23.3)
**Bacteria**	156 (59.5)
* Staphylococcus aureus*	52 (19.8)
Methicillin-susceptible *S.aureus*	5 (1.9)
Methicillin-resistant *S.aureus*	47(17.9)
* Acinetobacter baumannii*	42 (16.0)
* Pseudomonas aeruginosa*	29 (11.1)
* Klebsiella pneumoniae*	22 (8.4)
* Stenotrophomonas maltophilia*	12 (4.6)
* Escherichia coli*	6 (2.3)
* Enterobacter* species	5 (1.9)
* Streptococcus pneumoniae*	4 (1.5)
Unspecified *streptococcus* species	2 (0.8)
* Providencia stuartii*	1 (0.4)
* Proteus mirabilis*	1 (0.4)
* Moraxella catarrhalis*	1 (0.4)
* Legionella species*	1 (0.4)
* Klebsiella oxytoca*	1 (0.4)
**Virus**	59 (22.5)
Respiratory syncytial virus	16 (6.1)
Type A	11 (4.2)
Type B	5 (1.9)
Parainfluenza virus	16 (6.1)
Type 3	15 (5.7)
Type 1	2 (0.8)
Rhinovirus	15 (5.7)
Influenza virus	10 (3.8)
Influenza A	9 (3.4)
Influenza B	1 (0.4)
Cytomegalovirus	5 (1.9)
Human coronavirus	4 (1.5)
Human coronavirus OC43	2 (0.8)
Human coronavirus 229E/NL63	2 (0.8)
Bocavirus	2 (0.8)
Human metapneumovirus	2 (0.8)
Adenovirus	1 (0.4)
**Other**	
* Aspergillus* species	18 (6.9)
* Pneumocystis jirovecii*	6 (2.3)

HAP = hospital-acquired pneumonia.

a More than one pathogen was detected in some patients.

### Bacterial Pathogens

A total of 180 pathogens were identified in 156 patients. Two pathogens were detected in 22 patients, and three pathogens were detected in one patient. Of the 180 bacterial pathogens identified, 104 (66.7%) were detected from expectorated sputum cultures or endotracheal aspirate cultures, 38 (24.4%, one with *Legionella* PCR) were from BAL fluid cultures or PCRs, 24 (15.4%) were from blood cultures, four (2.6%, three with pneumococcal antigens, one with *Legionella* antigens) were detected via urinary antigens and three (1.9%) were from pleural fluid cultures. Eighteen patients (11.5%) had two or more positive tests. *S. aureus* was the most common pathogen (33.3%, 52/156), the majority of which were MRSA (90.4%, 47/52), followed by *A. baumannii* (26.9%, 42/156), *P. aeruginosa* (18.6%, 29/156), and *K. pneumoniae* (14.1%, 22/156).

### Respiratory Viral Pathogens

Overall, 71 viral pathogens were identified in 59 patients. Some of these patients were infected with more than one type of virus; two different pathogens were detected in eight of the patients (13.6%), and three pathogens were detected in two of the patients (3.4%). The distribution of viral pathogens for which specimens tested positive is shown in Table 3. Viruses were detected from BAL fluid specimens or the endotracheal aspirates of 37 patients (62.7%, 37/59) and solely from the nasopharyngeal specimens of 22 patients (37.3%, 22/59). In 19 patients (32.2%, 19/59), viruses were detected in both BAL fluid and nasopharyngeal samples and/or endotracheal aspirates.

**Table 3:** Microbiological diagnosis of respiratory viruses in hospital-acquired pneumonia.

Virus^a^	Total no. of Patients	BAL fluid ±endotrachealaspirate	Nasopharyngealspecimen only
	(n = 59) (%)	(n = 41) (%)	(n = 18) (%)
Respiratory syncytial virus	16 (27.1)	9 (22.0)	7 (38.9)
Type A	11 (18.6)	6 (14.6)	5 (27.8)
Type B	5 (8.5)	3 (7.3)	2 (11.1)
Parainfluenza virus	16 (27.1)	12 (29.3)	4 (22.2)
Type 3	15 (25.4)	11 (26.8)	4 (22.2)
Type 1	2 (3.4)	2 (4.9)	0
Rhinovirus	15 (25.4)	12 (29.3)	3 (16.7)
Influenza virus	10 (16.9)	8 (19.5)	2 (11.1)
Influenza A	9 (15.3)	7 (17.1)	2 (11.1)
Influenza B	1 (1.7)	1 (2.4)	0
Cytomegalovirus	5 (8.5)	5 (12.2)	0
Human coronavirus	4 (6.8)	4 (9.8)	0
Human coronavirus OC43	2 (3.4)	2 (4.9)	0
Human coronavirus 229E/NL63	2 (3.4)	2 (4.9)	0
Bocavirus	2 (3.4)	1 (2.4)	1 (5.6)
Human metapneumovirus	2 (3.4)	1 (2.4)	1 (5.6)
Adenovirus	1 (1.7)	1 (2.4)	0

BAL = bronchoalveolar lavage.

a Some cases were associated with two or more pathogens; two viruses were identified in eight patients and three viruses in two patients.

Respiratory syncytial virus and parainfluenza virus were the most commonly identified viruses (both 27.1% [16/59]), followed by rhinovirus (25.4%), influenza virus (16.9%), cytomegalovirus (8.5%), human coronavirus (6.8%), bocavirus (3.4%), human metapneumovirus (3.4%) and adenovirus (1.7%) (Table 2). The distribution of viral pathogens in patients with and without coinfection is shown in Table S2. Of the 59 patients with viral infection, 36 (61.0%) had coinfection with other organisms, including 21 (35.6%) with bacteria, 11 (18.6%) with another virus, and seven (11.9%) with fungus. The most commonly coexisting bacteria were *S. aureus* (n = 10) and *A. baumannii* (n = 9). The clinical characteristics and outcomes in patients with or without coinfection were not significantly different (Table S3). Table S4 summarizes the combinations of pathogens found in bacterial-viral coinfections. The most common combination was found to be respiratory syncytial virus A and *S. aureus* (n = 3), followed by respiratory syncytial virus B, *S. aureus* and *A. baumannii* (n = 2).

### Characteristics of 59 Patients with Viral Infection

The characteristics of the 59 patients with viral infections are summarized in Table 4. Among the 59 patients, the mean age was 59.3±15.4 years and the median duration of clinical symptoms before ICU admission was 3 days (IQR 2−5). The median length of hospital stay prior to pneumonia was 20 days (IQR 10−41). The majority of patients (94.9%, 56/59) had an underlying medical condition, including hematologic malignancy (45.8%, 27/59), structural lung disease (23.7%, 14/59) and diabetes mellitus (22.0%, 13/59). Forty-three patients were in an immunocompromised state (72.9%, 43/59). Radiographically, bilateral lung involvement was present in 57 patients (96.6%), and infiltration patterns are frequently diffuse (47.5%, 28/59) or multifocal (40.7%, 24/59). Ground-glass opacities were dominant in 19 patients (32.2%) (Table S3).

**Table 4:** Demographics, clinical characteristics, identity of pathogens, and outcomes of patients with severe pneumonia ^a^ ^,^ ^b^.

	Total (*n = 59*)	Immunocompromised c(n = 43)	Non-immunocompromised(n = 16)	*p*
Male, n (%)	39 (66.1)	28 (65.1)	11 (68.8)	0.793
Mean age (SD), y	59.3 (15.4)	55.8 (14.7)	68.6 (13.4)	0.004
Underlying disease, n (%)				
Hematologic malignancy	27 (45.8)	27 (62.8)	0	<0.001
Structural lung disease	14 (23.7)	9 (20.9)	5 (31.3)	0.407
Interstitial lung disease	7 (11.9)	7 (16.3)	0	0.086
Chronic obstructive pulmonary disease	4 (6.8)	1 (2.3)	3 (18.8)	0.026
Tuberculous destroyed lung	4 (6.8)	1 (2.3)	3 (18.8)	0.026
Diabetes mellitus	13 (22.0)	8 (18.6)	5 (31.3)	0.297
Bone marrow transplantation	10 (16.9)	10 (23.3)	0	0.034
Solid organ transplantation	6 (10.2)	6 (14.0)	0	0.115
Solid cancer	6 (10.2)	3 (7.0)	3 (18.8)	0.183
Cerebrovascular attack	4 (6.8)	2 (4.7)	2 (12.5)	0.286
Chronic kidney disease d	4 (6.8)	0	4 (25.0)	0.004
Hospital stay prior to pneumonia, median (IQR), d	20 (10–41)	22 (11–42)	17.5 (6.3–26.8)	0.302
Symptom duration before ICU admission, median (IQR), d	3.0 (2.0–5.0)	3.0 (2.0–5.0)	3.5 (2.0–5.5)	0.877
Clinical manifestation				
Fever (≥38.0°C)	44 (74.6)	32 (74.4)	12 (75.0)	0.964
Cough	36 (61.0)	28 (65.1)	8 (50.0)	0.290
Sputum	42 (71.2)	30 (69.8)	12 (75.0)	0.693
Dyspnea	51 (86.4)	36 (83.7)	15 (93.8)	0.317
Altered mentality	27 (45.8)	17 (39.5)	10 (62.5)	0.115
Shock	20 (33.9)	15 (34.9)	5 (31.3)	0.793
APACHE2 score, mean (SD)	25.6 (6.1)	26.4 (5.5)	23.1 (7.3)	0.08
SOFA score, mean (SD)	10.6 (3.3)	10.6 (3.4)	10.5 (3.0)	0.93
Viral pathogens, n (%)				
Respiratory syncytial virus	16 (27.1)	13 (30.2)	3 (18.8)	0.378
Parainfluenza virus	16 (27.1)	13 (30.2)	3 (18.8)	0.378
Rhinovirus	15 (25.4)	10 (23.3)	5 (31.3)	0.531
Influenza virus	10 (16.9)	5 (11.6)	5 (31.3)	0.074
Cytomegalovirus	5 (8.5)	5 (11.6)	0	0.154
Human coronavirus	4 (6.8)	2 (4.7)	2 (12.5)	0.286
Bocavirus	2 (3.4)	2 (4.7)	0	0.380
Human metapneumovirus	2 (3.4)	1 (2.3)	1 (6.3)	0.459
Adenovirus	1 (1.7)	1 (2.3)	0	0.538
Twenty-eight day mortality, n (%)	21 (35.6)	18 (41.9)	4 (25.0)	0.234

ICU = intensive care unit; APACHE = Acute Physiology and Chronic Health Evaluation; SOFA = Sequential Organ Failure Assessment; IQR = interquartile range.

a Categories of coinfection are not mutually exclusive. Some cases were associated with two or more pathogens.

b Cases of coinfection with organisms other than bacteria/other virus (six with *Aspergillus* species, four with *P. jirovecii*) are not presented in the Table.

c The immunocompromised state was verified if the patients: *(i)* had daily administration of corticosteroids (at least 5 mg per day of prednisolone or an equivalent drug), *(ii)* were solid organ or hematopoietic stem cell transplant recipients, *(iii)* had received treatment with chemotherapy for an underlying malignancy during the 6 months prior to ICU admission, and *(iv*) had an underlying acquired immune deficiency disorder.

d Includes two patients with chronic renal failure and two patients with end-stage renal disease.

Comparison of the 43 immunocompromised patients with the 16 non-immunocompromised patients is included in Table 4. Non-immunocompromised patients were significantly more likely to be older than immunocompromised patients and more commonly had underlying chronic obstructive pulmonary disease (COPD), tuberculous destroyed lung and chronic kidney disease. Although the mean APACHE II score upon ICU admission tended to be higher in non-immunocompromised patients (immunocompromised host, 26.4±5.5 vs. non-immunocompromised host, 23.1±7.3; *p* = 0.08), other clinical manifestations were similar between the two groups.

**Figure 2:**
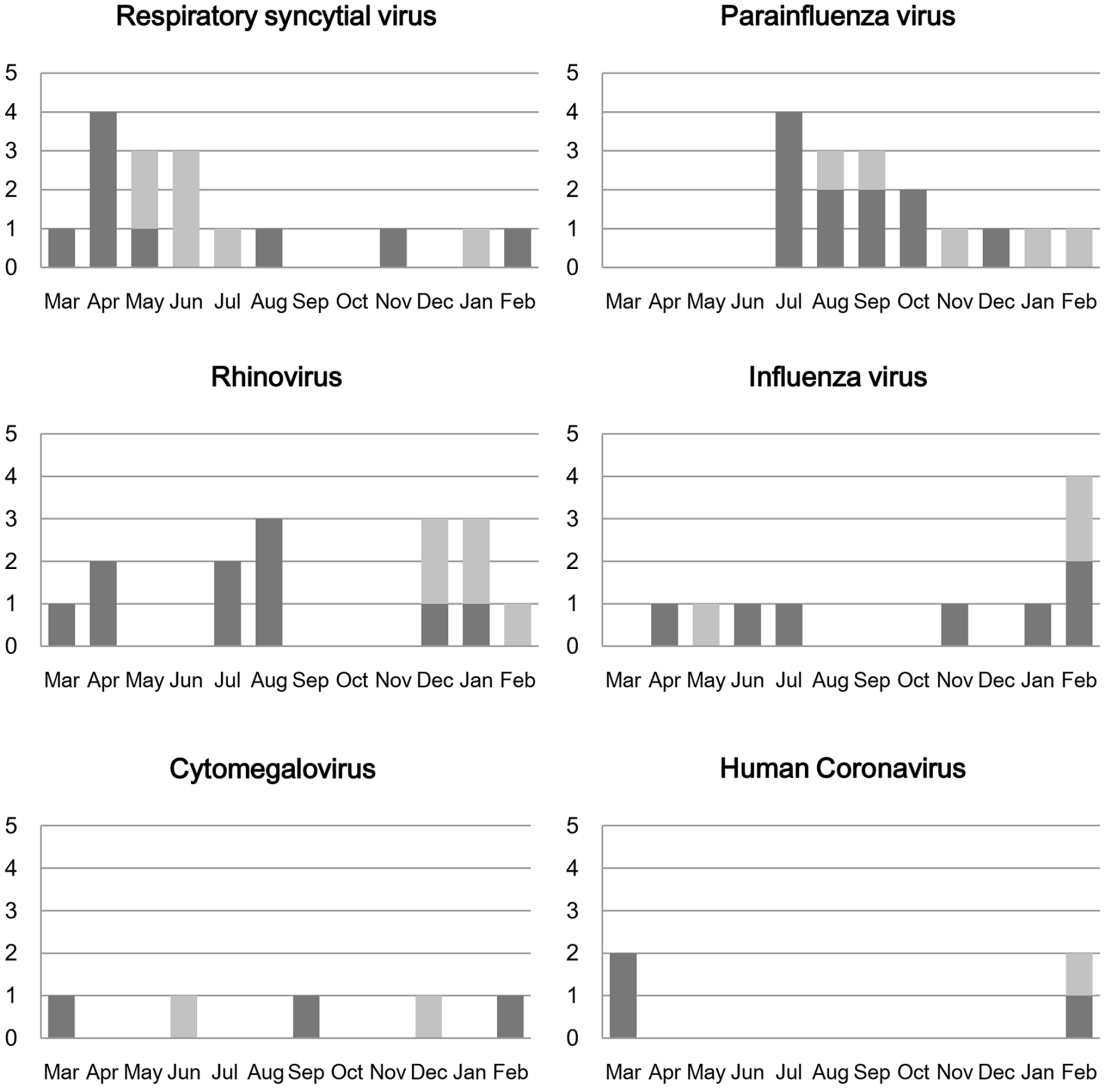
Monthly distribution of respiratory viruses detection. Black represents virus-positive cases in the first year and grey represents virus-positive cases in the second year.

Figure 2 shows the seasonality of respiratory virus infections during the study period. Respiratory syncytial virus was commonly found from April to August and parainfluenza virus predominated from July to December. Rhinovirus was present throughout the year but was more common in spring and winter. Influenza virus was mainly present in winter and human coronavirus was present from February to March.

### Outcomes

The results of our analysis of risk factors associated with 28 day mortality are given in Table 5. The overall 28 day mortality rate in the study population was 35.9% (94/262) and the in-hospital mortality rate was 49.2% (129/262). The median ICU stay was 12 days (IQR 7−20). The 28 day mortality rate was 29.5% (46/156) for bacterial infections, 30.8% (40/130) for sole bacterial infections, 35.6% (21/59) for viral infections, 38.7% (12/31) for sole viral infections and 19.0% (4/21) for bacterial-viral coinfections.

**Table 5:** Twenty-eight day mortality risk of patients with severe pneumonia.

Variable	No. of deaths/no. of episodes (%)	*p*	Adjusted Odds Ratio (95% CI)	*p*
All cases	94/262 (35.9)			
Sex				
Male	68/191(36.6)	0.879		
Female	26/71 (35.6)			
Shock at the time of admission		0.001	1.99 (1.15–3.46)	0.014
No	37/139 (26.6)			
Yes	57/123 (46.3)			
Structural lung disease		0.640		
No	74/202 (36.6)			
Yes	20/60 (37.1)			
COPD		0.396		
No	86/234 (36.8)			
Yes	8/28 (28.6)			
Interstitial lung disease		0.121	1.93 (0.70–5.34)	0.206
No	84/243 (33.3)			
Yes	10/19 (27.6)			
Diabetes mellitus		0.077	0.72 (0.39–1.35)	0.305
No	73/186 (38.6)			
Yes	21/76 (27.6)			
Solid cancer		0.330		
No	76/203 (37.4)			
Yes	18/59 (30.5)			
Hematologic malignancy		0.014	1.06 (0.53–2.11)	0.870
No	58/186 (33.3)			
Yes	36/76 (47.4)			
Immunocompromised state		<0.001	2.63 (1.51–4.60)	<0.001
No	34/143 (36.2)			
Yes	60/119 (63.8)			
McCabe and Jackson criteria		0.002	1.73 (0.93–3.21)	0.084
Nonfatal	23/97 (23.3)			
Rapidly fatal or ultimately fatal	71/165 (42.4)			
Pathogen identified		0.520		
No	24/61 (37.7)			
Yes	70/201 (34.8)			
Pathogen		0.321		
Bacteria only	40/130 (30.8)			
Virus(es) only	12/31 (38.7)			
Bacteria/virus coinfection	4/21 (19.0)			
*S. aureus*		0.593		
No	77/210 (36.7)			
Yes	17/52 (32.7)			
*P. aeruginosa*		0.077	0.47 (0.17–1.26)	0.131
No	88/233 (37.8)			
Yes	6/29 (20.7)			
Rhinovirus		0.198	0.38 (0.10–1.48)	0.119
No	91/247 (36.8)			
Yes	3/15 (20.0)			
Parainfluenza virus		0.500		
No	87/246 (35.4)			
Yes	7/16 (43.8)			
Influenza virus		0.501		
No	89/251 (35.5)			
Yes	5/11 (45.5)			
Respiratory syncytial virus		0.889		
No	88/246 (35.8)			
Yes	6/16 (37.5)			
Cytomegalovirus		0.472		
No	91/256 (35.5)			
Yes	3/6 (50.0)			
Human coronavirus		0.142	9.67 (0.85–109.49)	0.067
No	91/258 (35.3)			
Yes	3/4 (75.0)			

COPD = chronic obstructive pulmonary disease.

In multiple logistic regression analysis, independent predictors of 28 day mortality were associated with presence of shock (aOR 1.99, 95% CI 1.15 to 3.46, *p* = 0.01) and immunocompromised state (aOR 2.63, 95% CI 1.51 to 4.60, *p*<0.001).

## Discussion

The main finding of the present study is that viral infection is not uncommon in critically ill adult patients with severe HAP. Viruses were identified in 22.5% (59/262) of severe HAP patients, of whom 43 (72.9%) were immunocompromised and 16 (27.1%) were non-immunocompromised. Non-immunocompromised patients were older than immunocompromised patients and more commonly had COPD, tuberculous destroyed lung disease, and/or chronic kidney disease. Viral infection was associated with comparable mortality rates to bacterial infection. To our knowledge, this is the first study that was focused on respiratory viral pathogens in adult patients with severe HAP. The strength of our study is that viruses were detected from BAL fluid specimens in nearly two-thirds of our subject patients (62.7%, 37/59), which suggests that considerable numbers of identified viruses can be regarded as actual pathogens.

In our results, respiratory syncytial virus (27.1%), parainfluenza virus (27.1%) and rhinovirus (25.4%) were the most common viral pathogens, observed in 79.7% (47/59) of patients with viral infection. Prior studies regarding patients with hematologic malignancy or hematopoietic stem cell transplant, or solid organ transplants [9]–[13], also showed that respiratory syncytial virus, influenza virus and parainfluenza virus were major viral pathogens in those populations. Rhinovirus, a well-known cause of the common cold, is increasingly being recognized as an important cause of lower respiratory tract infection, mainly in severely immunocompromised patients, such as hematopoietic stem cell transplant patients [13], [29]. In our current study cohort, rhinovirus was found to be the third most commonly identified pathogen and to be more commonly associated with severe HAP compared with influenza virus. However, the significance of rhinovirus detection in respiratory specimens from pneumonia patients is a subject of some debate. Earlier studies have indicated that chronic shedding of rhinoviruses for more than 4 weeks is not uncommon in both immunocompetent individuals and immunocompromised patients [30], [31]. In our present study, rhinoviruses were identified in the BAL fluid from 10 out of 15 rhinovirus-positive cases, which suggests that, at least in these cases, these viruses would be significant pathogens rather than colonizers. In our present study, compared with our previous results regarding CAP or HCAP [23], the proportion of human metapneumovirus was found to be much lower in severe HAP cases (CAP or HCAP, 18.1% [13/72] vs. HAP, 3.4% [2/59]). Notably, viral infection in non-immunocompromised patients was not uncommon (11.1%, 16/143), although it was not as frequent as that in immunocompromised patients (36.4%, 43/119). Compared with immunocompromised patients, non-immunocompromised patients were older and more likely to have underlying COPD, tuberculous destroyed lung disease, and/or chronic kidney disease (Table 4). Taken together, our data suggest that, even in patients who are not known to be immunocompromised, viral infection should be considered when patients are elderly, especially in those with structural lung disease or chronic kidney disease.

The impact of coinfection with respiratory viruses and other organisms is an important issue. Prior studies of viral pneumonia in patients with hematologic malignancy or who received haematopoietic stem cell transplant have reported a lower incidence of coinfection ranging from 8.8% to 14.3% [6], [32]. In our present analyses, as found previously in adult CAP [23], coinfection was commonly identified. Of 59 patients with identified viral pathogens, 23 (39.0%) were infected by a single virus, 21 (35.6%) were coinfected with bacteria and 11 (16.9%) were coinfected with another virus. In CAP, some reports have shown that polymicrobial infection can be associated with poorer outcomes [33]–[37], most of these coinfections were influenza plus pneumococcal infections [36], [37]. In our results, however, clinical manifestation and outcomes in patients with or without coinfection were not significantly different (Table S2) and none of the combinations of polymicrobial pathogens were dominantly observed. The 28 day mortalities of our patients with bacterial infections, viral infections and bacterial-viral coinfections were 29.5% (46/156), 35.6% (21/59) and 19.0% (4/21) (*p* = 0.321), respectively. However, considering the relatively small number of patients with various combinations of pathogens, the severity of underlying disease and comorbidities, further investigation is required with larger populations.

The seasonal distribution of respiratory viral infections from HAP was very similar to that of infections from CAP [23]. That is, respiratory syncytial virus and influenza virus were frequent in winter and spring, parainfluenza virus was mainly present in summer to autumn, and rhinovirus was found throughout the year. This indicates that hospital-acquired viral infection occurs coincident with the spread of viral infection in the community. Therefore, watchful monitoring of community-level viral infection could be helpful for the prediction of hospital-acquired viral infection.

Our study has several limitations. First, the study was conducted at a single center and included only patients with severe pneumonia who were admitted to the medical ICU; thus, the results may not be generalizable. Second, since our study was an observational one, we could not intervene in terms of specific procedures or laboratory tests. Therefore, some patients did not undergo bronchoscopic BAL or testing for respiratory virus. This means that the actual rate of viral infection might be higher than that indicated by our results. Third, among 59 patients with viral infection, viruses were isolated solely from nasopharyngeal specimens in 22 patients (37.3%). These cases might include cases of coincidental upper respiratory viral infection. Finally, we did not perform virus testing for herpes simplex virus, which is known to cause pneumonia, especially in immunocompromised patients with hematologic malignancy [38].

In conclusion, viral pathogens are not uncommonly identified in adult patients with severe HAP who required intensive care. As these viruses could be responsible for severe HAP, and could represent a potential source of intra- or inter-hospital transmission, further investigation is required to delineate the prevalence and role of viral pathogens in severe HAP more precisely.

## Supporting Information

Table S1 Identification of viral pathogens according to the immunocompromised conditions. ^a^Some patients had two or more immunocompromised conditions. ^b^Some cases were associated with two or more viruses. ^c^Daily administration of corticosteroids at least 5 mg per day of prednisolone or an equivalent drug.
(DOC)

Table S2 Identity of viral pathogens in patients with and without coinfection. ^a^Categories of coinfection are not mutually exclusive. Some cases were associated with two or more pathogens. ^b^Cases of coinfection with organisms other than bacteria/other viruses (six with *Aspergillus* species, four with *P. jirovecii*) are not presented in the Table. ^c^The coexisting bacteria were *S. aureus* (n = 10), *A. baumannii* (n = 9), *S. maltophilia* (n = 2), *P. aeruginosa* (n = 2), *E. coli* (n = 1), *K. pneumoniae* (n = 1), and *S. pneumoniae* (n = 1).
(DOC)

Table S3 Demographics, clinical characteristics, identity of pathogens, and outcomes of patients with severe pneumonia and viral infection.**^a,b^**Abbreviations: ICU = intensive care unit, APACHE = Acute Physiology and Chronic Health Evaluation, SOFA = Sequential Organ Failure Assessment, IQR = interquartile range. **^a^**Categories of coinfection are not mutually exclusive. Some cases were associated with two or more pathogens. **^b^**Cases of coinfection with organisms other than bacteria/other viruses (six with *Aspergillus* s pecies, four with *P. jirovecii*) are not presented in the table.
(DOC)

Table S4 Combinations of pathogens in 21 patients with bacterial-viral coinfections.
(DOC)
