# Supplementary material for: Viral Infection Is Not Uncommon in Adult Patients with Severe Hospital-Acquired Pneumonia
Source: PLoS One. 2014 Apr 21;9(4):e95865. doi: 10.1371/journal.pone.0095865 (PMC3994115; doi:10.1371/journal.pone.0095865)
Supplement: Table S1 — Identification of viral pathogens according to the immunocompromised conditions. aSome patients had two or more immunocompromised conditions. bSome cases were associated with two or more viruses. cDaily administration of corticosteroids at least 5 mg per day of prednisolone or an equivalent drug. (DOC) [file pone.0095865.s001.doc]

**Table S1. Identification of viral pathogens according to the immunocompromised conditions**

| **Immunocompromised conditions^a^** | **Number of patients with identified respiratory virus/number of patients (%)** | **Respiratory virus (n) ^b^** |
| --- | --- | --- |
| Receipt of chemotherapy and/or radiation therapy in the previous 6 months | 21/57 (36.8) | Parainfluenza virus (9), respiratory syncytial virus (7), rhinovirus (4), bocavirus (2), influenza A (2), cytomegalovirus (1) |
| Receipt of corticosteroids^c^ | 20/56 (35.7) | Parainfluenza virus (5), respiratory syncytial virus (5), rhinovirus (5), cytomegalovirus (4), influenza A (3), human metapneumovirus (1), human coronavirus; (2), adenovirus. (1) |
| Receipt of non-steroidal immunosuppressant | 16/33 (48.5) | Parainfluenza virus (5), rhinovirus (5), respiratory syncytial virus (4), cytomegalovirus (2), influenza A (2), human metapneumovirus (1), human coronavirus (1) |
| Hematopoietic stem cell transplantation | 10/20 (50.0) | Parainfluenza virus (4), respiratory syncytial virus (3), rhinovirus (3), influenza A (2), bocavirus (1), human coronavirus; (1), human metapneumovirus (1) |
| Solid organ transplantation | 6/10 (60.0) | Cytomegalovirus (2), rhinovirus (2), human coronavirus; (1), influenza A (1), respiratory syncytial virus (1) |

^a^ Some patients had two or more immunocompromised conditions.

^b^ Some cases were associated with two or more viruses.

^c^ Daily administration of corticosteroids at least 5 mg per day of prednisolone or an equivalent drug.
