# Supplementary material for: Viral Infection Is Not Uncommon in Adult Patients with Severe Hospital-Acquired Pneumonia
Source: PLoS One. 2014 Apr 21;9(4):e95865. doi: 10.1371/journal.pone.0095865 (PMC3994115; doi:10.1371/journal.pone.0095865)
Supplement: Table S2 — Identity of viral pathogens in patients with and without coinfection. aCategories of coinfection are not mutually exclusive. Some cases were associated with two or more pathogens. bCases of coinfection with organisms other than bacteria/other viruses (six with Aspergillus species, four with P. jirovecii) are not presented in the Table. cThe coexisting bacteria were S. aureus (n = 10), A. baumannii (n = 9), S. maltophilia (n = 2), P. aeruginosa (n = 2), E. coli (n = 1), K. pneumoniae (n = 1), and S. pneumoniae (n = 1). (DOC) [file pone.0095865.s002.doc]

**Table S2. Identity of viral pathogens in patients with and without coinfection**

|  | | **Total (n=59) (%)** | **Coinfection with bacteria^c^ (n=21)** | **Coinfection with another virus (n=11)** | **No coinfection (n=23)** |
| --- | --- | --- | --- | --- | --- |
| **Virus** **^a,b^** | |
| Respiratory syncytial virus | | 16 (27.1) | 6 | 4 | 7 |
|  | Type A | 11 (18.6) | 4 | 3 | 5 |
|  | Type B | 5 (8.5) | 2 | 1 | 2 |
| Parainfluenza virus | | 16 (27.1) | 6 | 5 | 4 |
|  | Type 3 | 15 (25.4) | 5 | 5 | 4 |
|  | Type 1 | 2 (3.4) | 1 | 1 | 0 |
| Rhinovirus | | 15 (25.4) | 5 | 5 | 5 |
| Influenza virus | | 10 (16.9) | 3 | 2 | 5 |
|  | Influenza A | 9 (15.3) | 3 | 2 | 4 |
|  | Influenza B | 1 (1.7) | 0 | 0 | 1 |
| Cytomegalovirus | | 5 (8.5) | 2 | 2 | 0 |
| Human coronavirus | | 4 (6.8) | 0 | 3 | 0 |
|  | Human coronavirus OC43 | 2 (3.4) | 0 | 2 | 0 |
|  | Human coronavirus 229E/NL63 | 2 (3.4) | 0 | 1 | 0 |
| Bocavirus | | 2 (3.4) | 0 | 1 | 1 |
| Human metapneumovirus | | 2 (3.4) | 0 | 1 | 1 |
| Adenovirus | | 1 (1.7) | 0 | 0 | 0 |

^a^ Categories of coinfection are not mutually exclusive. Some cases were associated with two or more pathogens.

^b^ Cases of coinfection with organisms other than bacteria/other viruses (six with *Aspergillus* species, four with *P. jirovecii*) are not presented in the Table.

^c^ The coexisting bacteria were *S. aureus* (n=10), *A. baumannii* (n=9), *S. maltophilia* (n=2), *P. aeruginosa* (n=2), *E. coli* (n=1), *K. pneumoniae* (n=1), and *S. pneumoniae* (n=1).
