# Supplementary material for: Viral Infection Is Not Uncommon in Adult Patients with Severe Hospital-Acquired Pneumonia
Source: PLoS One. 2014 Apr 21;9(4):e95865. doi: 10.1371/journal.pone.0095865 (PMC3994115; doi:10.1371/journal.pone.0095865)
Supplement: Table S3 — Demographics, clinical characteristics, identity of pathogens, and outcomes of patients with severe pneumonia and viral infection.a,bAbbreviations: ICU = intensive care unit, APACHE = Acute Physiology and Chronic Health Evaluation, SOFA = Sequential Organ Failure Assessment, IQR = interquartile range. aCategories of coinfection are not mutually exclusive. Some cases were associated with two or more pathogens. bCases of coinfection with organisms other than bacteria/other viruses (six with Aspergillus s pecies, four with P. jirovecii) are not presented in the table. (DOC) [file pone.0095865.s003.doc]

**Table S3. Demographics, clinical characteristics, identity of pathogens, and outcomes of patients with severe pneumonia and viral infection a,b**

|  | | **Total (*n=59*)** | **Coinfection with bacteria (*n=21*)** | **Coinfection with another virus (*n=11*)** | **No coinfection (*n=23*)** |
| --- | --- | --- | --- | --- | --- |
| Male, n (%) | | 39 (66.1) | 14 (66.7) | 9 (81.8) | 13 (56.5) |
| Mean age (SD), y | | 59.3 (15.4) | 58.6 (18.0) | 63.5 (8.3) | 56.4 (14.8) |
| Underlying disease, n (%) | |  |  |  |  |
|  | Hematologic malignancy | 27 (45.8) | 8 (38.1) | 5 (45.5) | 13 (56.5) |
|  | Structural lung disease | 14 (23.7) | 7 (33.3) | 2 (18.2) | 2 (8.7) |
|  | Diabetes mellitus | 13 (22.0) | 5 (23.8) | 4 (36.4) | 2 (8.7) |
|  | Bone marrow transplantation | 10 (16.9) | 2 (9.5) | 4 (36.4) | 4 (17.4) |
|  | Solid cancer | 6 (10.2) | 2 (9.5) | 1 (9.1) | 3 (13.0) |
|  | Solid organ transplantation | 6 (10.2) | 3 (14.3) | 1 (9.1) | 2 (8.7) |
|  | Immunocompromised state | 43 (72.9) | 14 (66.7) | 8 (72.7) | 17 (73.9) |
|  | Receipt of immunosuppressant therapy | 25 (44.1) | 8 (38.1) | 5 (45.5) | 8 (34.8) |
|  | Receipt of recent chemotherapy (within 1 month) | 21 (33.9) | 5 (23.8) | 3 (27.3) | 12 (52.3) |
|  | Neutropenia (absolute neutrophil count < 500/mm^3^) | 15 (27.1) | 5 (23.8) | 2 (18.2) | 7 (30.4) |
| Hospital stay prior to pneumonia, median (IQR) | | 20 (10–41) | 20 (5.5–43.5) | 23 (18–31) | 17 (10–24) |
| Symptom duration before ICU admission, median (IQR) | | 3.0 (2–5) | 4.0 (1.5–5.5) | 3.0 (1–5) | 4.0 (2–5) |
| Clinical manifestation, n (%) | |  |  |  |  |
|  | Fever (≥ 38.0°C) | 44 (74.6) | 16 (76.2) | 8 (72.7) | 17 (73.9) |
|  | Cough | 36 (61.0) | 12 (57.1) | 7 (63.6) | 14 (60.9) |
|  | Sputum | 42 (71.2) | 16 (76.2) | 10 (90.9) | 14 (60.9) |
|  | Dyspnea | 51 (86.4) | 17 (81.0) | 10 (90.9) | 20 (87.0) |
|  | Altered mentality | 27 (45.8) | 10 (47.6) | 5 (45.5) | 11 (47.8) |
|  | Shock | 20 (33.9) | 5 (23.8) | 3 (27.3) | 11 (47.8) |
|  | APACHE2 score, mean (SD) | 25.6 (6.1) | 24.9 (5.9) | 28.3 (5.1) | 25.7 (6.9) |
|  | SOFA score, mean (SD) | 10.6 (3.3) | 10.9 (3.0) | 10.3 (2.7) | 11.1 (3.6) |
| Laboratory findings, median (IQR) | |  |  |  |  |
|  | White blood cells (/mm^3^) | 6,000 (1,000–11,700) | 6,600 (1,100–12,250) | 2,600 (1,000–10,300) | 6,600 (700–13,000) |
|  | Hemoglobin(g/dL) | 9.2 (8.2–10.5) | 9.7 (7.8–10.8) | 9.2 (8.2–10.3) | 9.1 (8.2–10.8) |
|  | Platelets (10^3^/mm^3^) | 88 (36–206) | 113 (38–210) | 52 (36–89) | 74 (36–137) |
|  | C-reactive protein (mg/dL) | 15.9 (8.5–23.5) | 9.6 (5.2–24.0) | 20.3 (9.4–25.2) | 16.3 (12.2–26.0) |
|  | Procalcitonin (ng/mL) | 1.08 (0.45–9.37) | 1.67 (0.40–12.28) | 1.70 (0.88–2.05) | 1.13 (0.41–12.15) |
|  | Lactate dehydrogenase (IU/L) | 315 (187–420) | 314 (140–448) | 277 (179–374) | 384 (264–570) |
| Radiologic findings, n(%) | |  |  |  |  |
|  | Bilateral involvement | 57 (96.6) | 19 (90.5) | 11 (100) | 23 (100) |
|  | Diffuse involvement | 28 (47.5) | 6 (28.6) | 6 (54.5) | 13 (56.5) |
|  | Multifocal consolidations | 24 (40.7) | 12 (57.1) | 3 (27.3) | 9 (39.1) |
|  | Ground-glass opacities | 19 (32.2) | 4 (19.0) | 5 (45.5) | 5 (21.7) |
|  | Pleural effusion | 6 (10.2) | 2 (9.5) | 3 (27.3) | 1 (4.3) |

ICU = intensive care unit; APACHE = Acute Physiology and Chronic Health Evaluation; SOFA = Sequential Organ Failure Assessment; IQR = interquartile range.

**^a^**: Categories of coinfection are not mutually exclusive. Some cases were associated with two or more pathogens.

**^b^**: Cases of coinfection with organisms other than bacteria/other viruses (six with *Aspergillus* species, four with *P. jirovecii*) are not presented in the table.
