# Supplementary material for: Viral Infection Is Not Uncommon in Adult Patients with Severe Hospital-Acquired Pneumonia
Source: PLoS One. 2014 Apr 21;9(4):e95865. doi: 10.1371/journal.pone.0095865 (PMC3994115; doi:10.1371/journal.pone.0095865)
Supplement: Table S4 — Combinations of pathogens in 21 patients with bacterial-viral coinfections. (DOC) [file pone.0095865.s004.doc]

**Table S4. Combinations of pathogens in 21 patients with bacterial-viral coinfections.**

| **Pathogens** | **Subjects (n)** |
| --- | --- |
| Respiratory syncytial virus A +*S. aureus* | 3 |
| Respiratory syncytial virus A + rhinovirus + *A. baumannii* | 1 |
| Respiratory syncytial virus B + *A. baumannii + S. aureus* | 2 |
| Parainfluenza virus-3 + *K. pneumoniae + S. aureus* | 1 |
| Parainfluenza virus-3 + *E. coli* | 1 |
| Parainfluenza virus-3 + *P. aeruginosa* | 1 |
| Parainfluenza virus-3 + *S. aureus* | 1 |
| Parainfluenza virus-3 + *S. maltophilia* | 1 |
| parainfluenza virus-1 + *A. baumannii* | 1 |
| Rhinovirus + *A. baumannii + S. aureus* | 1 |
| Rhinovirus + *A. baumannii* | 1 |
| Rhinovirus + *S. aureus* | 1 |
| Rhinovirus + *S. maltophilia* | 1 |
| Influenza A + *A. baumannii + P. aeruginosa* | 1 |
| Influenza A + *A. baumannii* | 1 |
| Influenza A + *S. aureus* | 1 |
| Cytomegalovirus *+ A. baumannii* | 1 |
| Cytomegalovirus + *S. pneumonia* | 1 |
